# Hope, happiness and home treatment: a study into patient satisfaction with being treated at home

**DOI:** 10.1192/pb.bp.112.040188

**Published:** 2014-12

**Authors:** Dieneke Hubbeling, Robert Bertram

**Affiliations:** 1 Springfield University Hospital, London

## Abstract

**Aims and method** This study investigates patient satisfaction and levels of hope after receiving treatment from a home treatment team. It studies whether distributing questionnaires during the last visit increases the response rate, and explores whether patient satisfaction and levels of hope are associated with particular elements of the care received.

**Results** Patients who answered the questionnaire tended to be satisfied. When forms were distributed during the last visit, the response rate increased to at least 64%. People with negative views were more likely to return the form by post. Patient satisfaction and levels of hope were associated with most elements of received care, and the resolution of problems was predictive of both satisfaction and increased hope in logistic regression.

**Clinical implications** The distribution of service evaluation questionnaires during the last visit increased the response rate considerably. This study suggests that in order to improve services, it is important to focus on whether patients think their problems have been resolved.

Crisis resolution and home treatment teams (CRHTs) were introduced in the UK in 2000 to reduce in-patient bed use. They were also intended to produce similar or improved results, in terms of both symptomatic outcome and patient satisfaction. Although there is one published randomised controlled trial which favoured home treatment,^1^ the evidence for CRHTs is still rather limited.^2,3^ Furthermore, there are considerable differences in local funding arrangements and services provided,^4^ because the guidelines for CRHTs published in the *Mental Health Policy Implementation Guide* are not mandatory.^5^ It is therefore essential that we evaluate local service provision, including patient-reported outcomes.

There are considerable challenges in performing local service evaluations. There is no agreement about how patient satisfaction should be evaluated in general,^6^ let alone in the context of CRHTs. Furthermore, response rates can be so low that it is difficult to draw any conclusions.^2,3^ This study explores the usefulness of a simple protocol in measuring service satisfaction with CRHTs to see whether meaningful data can be obtained using an enhanced recruitment procedure. Our aim was to discover the proportion of users of the Wandsworth Crisis and Home Treatment Team who feel that the service is of benefit to them. Further, we aimed to obtain patients’ help in identifying aspects of the service that could be improved and we hoped to be able to make links between our service provision and any restrictions in resources and levels of patient satisfaction.

## Method

### Setting and service configuration

Wandsworth is a London borough with approximately 285 000 residents, including some relatively wealthy and many relatively deprived areas. During the period covered by this study (1 July to 1 November 2011), the Wandsworth Crisis and Home Treatment Team offered home visits between 09:00 h and 21:00 h, and telephone contact outside these hours. Patients were adults aged 18 or over (there was no upper age limit) who were experiencing a mental health problem severe enough that it would otherwise have resulted in hospital admission. They could stay with the team for up to 4 weeks; after 4 weeks, they were either referred to a community mental health team or primary care, or admitted to hospital, depending on symptoms, risk and social circumstances. Only in exceptional circumstances could patients stay on longer than 4 weeks: examples may include a patient on antipsychotic medication who is showing a marked improvement but is having problems with adherence. The maximum treatment period of 4 weeks is one way in which the team had to deviate from standard practice for funding reasons as staffing levels were approximately 50% of those recommended by the Department of Health.^5^ Another such deviation was not being able to offer a designated named worker, which is a requirement of the Royal College of Psychiatrists’ accreditation scheme.^7^

Given the funding situation, service evaluation from a patient perspective was essential. Patients were asked whether they were satisfied with the care received, and whether it had made them more hopeful about the future. The National Collaborating Centre for Mental Health advised that satisfaction with care should be regularly surveyed, along with other aspects of patient experience.^8^ Yalom emphasised the importance of hope as a curative factor in psychotherapy,^9^ but it is also an important element of recovery,^10^ which nevertheless does not seem to be clearly assessed by standard clinical outcome measures.^11^ Apart from assessing patient satisfaction and levels of hope, the study also aimed to explore whether these factors were associated with specific aspects of the care offered by the CRHT (e.g. the length of the visits), because this would give a clear indication of the focus for service improvement. The questionnaire had to be brief and easy to fill in, given the low response rates in other similar studies.^2,3^ An unpublished pilot study with an extensive postal questionnaire conducted in 2008 had a response rate of less than 10%.

### Questionnaire

The Client Satisfaction Questionnaire^12^ is brief and easy to fill in, but it does not relate patient satisfaction to specific aspects of the care received. There are more extensive scales for measuring patient satisfaction and the care received, such as the Verona Service Satisfaction Scale,^13^ but none are specifically geared towards CRHTs. We therefore decided to develop our own questionnaire and to use the same overall question on patient satisfaction as was used in the Care Quality Commission’s national survey.^14^ Patients were asked ‘What is your overall view of the service from the Home Treatment Team?’, and could answer ‘excellent’, ‘very good’, ‘good’, ‘fair’, ‘poor’ or ‘very poor’. When this study began there was no standard question available for uncovering levels of hope, so we constructed the question, ‘How hopeful are you that you will be able to do the things you want to do in life?’. We also developed questions regarding aspects of the care received, namely whether patients had been informed about the CRHT by the referrer, whether they received a written care plan, whether they received written information about CRHT treatment, whether there was enough time during visits, how many weeks they stayed with the CRHT, whether this length of time was appropriate, and whether their problems were resolved. Some patients told us that, after they had received the previous postal questionnaire, they had been worried about giving their demographic details because they feared that this would allow them to be identified. Demographic questions were therefore not included, but patients were asked to tell us about their diagnosis. Open questions about what was good or bad about the home treatment team were also added (see the online supplement to this paper for the full questionnaire).

The final questionnaire had good face validity when shown to the multidisciplinary team. A pilot was conducted in 2010 with over 50 patients and they were specifically asked for their views on the questionnaire; however, this question did not often receive a response (8 responses in total), and if patients did answer the question they wrote that it was ‘good’ or ‘OK’. Like Kumar & McBride,^15^ we found it difficult to actively engage patients in the development of the questionnaire. The way we developed the questionnaire clearly had limitations and might have introduced various biases, for example because we only tested face validity of the questionnaire with members of our own team and patients from our own catchment area. Because we were very concerned about the low response rate in the previous postal questionnaire, we distributed the questionnaire to patients at their discharge visit: they could choose to fill it in there and then (the member of staff was advised to wait in the corridor or another room) or to return it to the audit office in a stamped addressed envelope provided.

The specific hypotheses of the study were that we could increase the response rate by this enhanced recruitment procedure of asking patients to fill in the form at the last visit and that patients who returned the questionnaire by post would, on average, be less satisfied with the services and less hopeful about the future, and would have lower scores on received care, in other words any negative information would make it more likely that patients preferred to return the form by post. Following Perneger,^16^ we did not correct for multiple testing, and the results were analysed with IBM SPSS 22 using non-parametric tests because of the non-normal distribution.

## Results

A total of 152 responses were received between 1 July and 1 November 2011. The response rate could only be established by approximation, as responses were completely anonymised. It was at least 64%, because the number of crisis episodes (i.e. patients under the care of the team) in that period was 266, and the number of admissions to hospital after an initial period of home treatment was approximately 30. Patients who had two or more crisis episodes in this period were not excluded and might have filled in the questionnaire twice, commenting on different periods with the home treatment team. Patients who were admitted to hospital after an initial period of home treatment were not given the questionnaire. The minimum response rate was therefore 152/(266–30) = 0.64. However, we do not know how often staff forgot to give the patient the form, and this might have had a negative effect on the response rate. Of the people who returned the form, only 68 wrote anything about their diagnosis. It was therefore impossible to look for associations between diagnosis, patient satisfaction and levels of hope. In general, patients were satisfied with the service: 45% thought the service was excellent, 31.5% thought it was very good and 16.1% thought it was good.

### Giving form to staff *v.* sending it

Unfortunately, for 15 of the 152 responses it was not recorded whether they were posted to the audit office or handed to a member of staff, so only 137 forms could be analysed. Of the 137 remaining forms, 42 were sent directly to the audit office and 95 were given to a member of staff. The answers of both groups are reported in Table 1. Overall, as predicted, patients who returned the form by post were less satisfied with their treatment and less likely to consider their problems resolved. There was no statistically significant difference in levels of hope about the future, and we assumed that satisfied patients would be more hopeful about the future and thereby more likely give the questionnaire to a member of staff instead of posting it.

### Satisfaction

Three respondents did not answer the question on their overall view of their care; so 149 forms could be analysed: 114 patients responded by rating the care as excellent or very good, and 35 reported that it was good, fair, poor or very poor. The answers to the questions on the care received were then compared between these two groups (Table 2). This showed that all elements of care were associated with higher patient satisfaction, apart from whether patients were informed beforehand.

A backward conditional logistic regression model with all seven variables about the care received gave a model with two variables – namely, having received a written care plan and whether problems were resolved – significant at the *P*<0.05 level.

**Table 1:** Satisfaction and method of submission

	Questionnaire given to staff, *n* (%)	Questionnaire posted to the audit office, *n* (%)	χ^2^^a^
Reported received care^b^			
Informed beforehand	82 (86)	35 (83)	0.208
Written information	76 (80)	26 (62)	5.014*
Written care plan	45 (47)	7 (17)	11.659**
Enough time to discuss	81 (85)	28 (67)	6.194*
Shorter than 2 weeks	51 (54)	25 (60)	0.402
Right time with home treatment	76 (80)	30 (71)	1.22
Problems solved	74 (78)	24 (57)	6.159*
			
Overall view^c^			
More hope	54 (57)	20 (49)	0.750
More satisfied	78 (83)	24 (59)	9.234**

a. d.f. = 1.

b. Questionnaire given to staff, *n* = 95; questionnaire posted to audit office, *n* = 42.

c. Questionnaire given to staff, *n* = 95; questionnaire posted to audit office, *n* = 41.

* *P*<0.05

** *P*<0.01.

**Table 2:** Satisfaction and perception of received care

Reporter received care	More satisfied (*n* = 114), *n* (%)	Less satisfied (*n* = 35), *n* (%)	χ^2^^a^
Informed beforehand	96 (84)	28 (80)	0.340
			
Written information	89 (78)	21 (60)	4.525*
			
Written care plan	49 (43)	6 (17)	7.678**
			
Enough time to discuss	93 (82)	21 (60)	6.938**
			
Shorter than 2 weeks	58 (51)	26 (74)	5.966*
			
Right time with home treatment	96 (84)	23 (66)	5.697*
			
Problems solved	86 (75)	18 (51)	7.323**

a. d.f. = 1.

* *P*<0.05

** *P*<0.01.

### Hope

The results on respondents’ levels of hope are presented in Table 3. Because 13 respondents did not answer the question about hope, this leaves 139 responses for analysis: 78 patients answered that they were more hopeful about the future and 61 were less hopeful or the same as before home treatment team referral. Overall, the results relating to hope were fairly similar to those on patient satisfaction, apart from the fact that there was no significant difference for having a written care plan and whether people stayed the right time with the home treatment team.

A backward conditional logistic regression model with the seven variables of care received gave a model with two variables was significant at the *P*<0.01 level: having received written information and whether problems were resolved.

### Open questions

Overall, 117 patients responded to the open questions, and many said that the service was good, but there were some unexpected responses: one patient remarked that it was ‘good that I could smoke’, and another said that ‘my children were sometimes afraid of people coming’. A negative comment which was made six times was that patients were seen by too many different people; however, one patient wrote that it was ‘good to have different views’. Twelve patients said that they would have liked to have more specific visiting times, with one person writing: ‘I

**Table 3:** Hope and perception of received care

Reported received care	More hope (*n* = 78), *n* (%)	No more hope *(n* = 61), *n* (%)	χ^2^^a^
Informed beforehand	69 (88)	49 (80)	1.766
			
Written information	67 (86)	36 (59)	12.88**
			
Written care plan	34 (44)	18 (30)	2.899
			
Enough time to discuss	71 (91)	40 (66)	13.785**
			
Shorter than 2 weeks	38 (49)	40 (66)	3.949*
			
Right time with home treatment	66 (85)	44 (72)	3.231
			
Problems solved	68 (87)	33 (54)	18.858**

a. d.f. = 1.

* *P*<0.05

** *P*<0.01.

think you should be given appointment slots and not have to be at home waiting as they can come at any time’. Nobody mentioned that they would like to have had a written care plan from the CRHT, and actually the majority of the patients stated that they had not received one.

## Discussion

In the previous section we have presented the results of a service evaluation study, where patients were given questionnaires during their last visit and could choose to fill in the form there and then or to post it to the audit office.

In service evaluation studies, there is the problem of low response rates, unless research assistants are available to individually approach patients.^1^ Regarding home treatment teams, one study based on postal questionnaires had a response rate of 14.5% in the home treatment team group and 11% in a control group,^17^ whereas another study received an overall response rate of 29%.^18^ The response rate of the South West London and St George’s Mental Health NHS Trust in the Care Quality Commission’s 2011 postal community mental health survey was 28%,^14^ and it was considerably higher in the present study. Although our response rates increased to over 60%, we faced the disadvantage that we did not obtain patients’ demographic details or adequate information about their diagnoses. Patients told us that they were concerned about being recognised on the basis of their age and gender; perhaps they were also worried that they would be identifiable by their diagnosis. This study illustrates that there is a tradeoff between the response rate and the information gathered; however, it does provide some evidence that many patients are reasonably satisfied with the service.

There is no standard scale for assessing satisfaction or levels of hope in CRHTs, and there is no consensus on how to evaluate patient experience in this context. Patient satisfaction may intuitively seem like a plausible measure of the outcome of mental health treatment, but there are difficulties. Data in satisfaction studies tend to be skewed, as most patients report that they are quite satisfied. Patient satisfaction depends not only on diminishing symptoms and actual care received, but also on prior expectations.^19^ Maybe many people were satisfied in this study because they did not know what to expect and were only able to compare their treatment to experiences of hospital admission.

The overall satisfaction question in our questionnaire was similar to the one used in national surveys, so we could compare the results and counterbalance some of the difficulties with using a non-validated scale. Patients who sent the form to the audit office were less satisfied on average than those who handed it to staff, although 24 out of 41 patients (59%) still rated the service as excellent or very good. In the national postal survey for community mental health teams in the same time period, 59% rated their service as excellent or very good, and the South West London and St George’s Mental Health NHS trust scored within the average range for overall satisfaction.^14^ It appears that the responses from the patients who posted the form in this study are the same as the results of the national postal survey. Current data offer some support for the view that patients who are satisfied with services did not return the postal questionnaire form from the Care Quality Commission. However, we cannot exclude the possibility that some people might have felt pressured to give a positive response or that the response was so positive because patients were asked at discharge, leaving no time interval between discharge and the satisfaction questionnaire, whereas patients are more likely to be satisfied with their treatment immediately after discharge.^20^ Another major limitation of the study was that patients who were admitted to hospital after an initial period of home treatment were not included and there is a strong possibility that as the CRHT intervention was not successful for this group of patients, the feedback from them would have been less positive and may have significantly altered the findings of the study.

In this study, higher patient satisfaction scores were associated with the perception of having received various elements of care, as predicted. The one exception was whether patients were informed beforehand. This is somewhat counterintuitive: one might expect that patients would not appreciate unannounced visits. This result needs to be confirmed in other independent studies before firm conclusions can be drawn.

Like patient satisfaction, hope can mean different things to different people.^21^ However, most descriptions include a sense of hope that one can live a satisfying life within the limitations caused by the illness. Contrary to expectations, there was no statistically significant difference in levels of hope about the future between patients who returned the form by post and patients who gave the form to a member of staff. Otherwise, the results for levels of hope were more or less similar to those for patient satisfaction. The logistic regression results for both hope and satisfaction point to the importance of resolving problems, and this is something which should be focused on in the future.

This study suggests that interesting results can be obtained by giving forms to patients at the last visit and asking them to fill it in there and then. By giving them the option of posting it, we can at last partially correct for the fact that people with a negative judgement are less likely to give it to a member of staff. However, this study also confirms that it is difficult to measure patient satisfaction and levels of hope with a view to improving services, as the majority of patients will say that they are satisfied and hopeful. This study provides some preliminary evidence that it is most important for CRHTs to focus on making patients feel that their problems have been resolved.
